# #EntreViagenseAprendizagens: study protocol of a school-based intervention to promote well-being and healthy lifestyles among adolescents

**DOI:** 10.3389/fpsyg.2023.1213293

**Published:** 2023-07-17

**Authors:** Rita Francisco, Beatriz Raposo, Mafalda Hormigo, Mónica Sesifredo, Ana Carvalho, Ana Justo, Cristina Albuquerque Godinho

**Affiliations:** ^1^Católica Research Centre for Psychological - Family and Social Wellbeing (CRC-W), Universidade Católica Portuguesa, Lisbon, Portugal; ^2^School of Human Sciences, Universidade Católica Portuguesa, Lisbon, Portugal; ^3^NOVA National School of Public Health, Public Health Research Centre, Comprehensive Health Research Center (CHRC), NOVA University Lisbon, Lisbon, Portugal

**Keywords:** well-being, healthy lifestyles, social and emotional skills, school-based intervention, adolescents, experimental design, protocol

## Abstract

**Background:**

Adolescence is a critical period of development in which well-being usually decreases, mental health problems (e.g., depression, anxiety) increase, and lifestyles become less healthy. Schools are a primary setting for the promotion of the well-being and overall health of adolescents, and preventive actions should be a priority within the scope of health-promoting schools. #EntreViagenseAprendizagens is a school-based intervention aiming to promote well-being and healthy lifestyles among adolescents based on social and emotional learning, positive psychology, and health education approaches.

**Methods:**

This protocol describes a school-based intervention, #EntreViagenseAprendizagens, that will be implemented in several schools in Portugal. The program is aimed at 8th and 9th grade students (14–16 years old) and comprises 20 weekly sessions. One of the sessions is aimed at the students’ parents/guardians. The intervention content targets social and emotional skills, health literacy (physical and mental health), healthy lifestyles, character strengths, and well-being. An experimental design will be used in the intervention evaluation. Eighth grade classes will be randomly assigned to the intervention group or the control group. All students complete the same assessment protocol at baseline, post-intervention, and 9-month follow-up. The impact assessment protocol includes measures related to well-being, health literacy, health-related knowledge, attitudes and behaviors, relationships with others, social and emotional skills, and sociodemographic data. Process evaluation includes evaluation forms at the end of each session and at the end of the program and focus groups with students, parents, and teachers at the end of the program.

**Discussion:**

This school-based intervention may play an important role in promoting students’ well-being and in preventing unhealthy lifestyles and socio-emotional maladjustment, by focusing on the development of social and emotional skills and health literacy among adolescents, empowering them to face the changing future and grow up healthy. Furthermore, this project aims to provide relevant scientific findings that can contribute to the development of better health-promoting schools.

## 1. Introduction

In their work on health-promoting schools, World Health Organization and United Nations Educational, Scientific and Cultural Organization (2021), have stressed that preventive actions should be, more than ever, a priority for schools, in order to foster the well-being and overall health of children and adolescents in a safe learning environment. Although the relationship between well-being, physical health, and mental health is well-established, prevention programs implemented in school settings tend to focus on only one of these three dimensions, particularly in Portugal. Therefore, a school-based intervention named #EntreViagenseAprendizagens was developed to promote well-being and healthy lifestyles among adolescents based on social and emotional learning, positive psychology, and health education approaches. This study protocol aims to describe the research design and methodology of #EntreViagenseAprendizagens.

### 1.1. Health, well-being, and lifestyles during adolescence

Adolescence is a critical period of development in which well-being usually decreases (e.g., Hendriks et al., 2020; Orben et al., 2022), mental health problems (e.g., depression, anxiety, behavioral problems) increase (e.g., Leadbeater et al., 2012; Frey et al., 2020), and lifestyles become less healthy (e.g., Gunnell et al., 2016; Irvine et al., 2022), with significant implications during adulthood (e.g., Frech, 2012; Velten et al., 2018).

The relationship between lifestyles, physical health and mental health has been clearly identified in the literature, particularly among adolescents, as the Health Behavior in School-aged Children (HBSC) studies have shown (e.g., Marques et al., 2019). For example, the longitudinal study developed by Gunnell et al. (2016) found that higher psychopathology symptoms were associated with more screen time and less physical activity, and that higher initial symptoms of depression predicted greater decreases in physical activity during adolescence. Also, Ames and Leadbeater (2018) identified different developmental trajectories from adolescence to young adulthood, which relate to depressive symptoms and specific indicators of health (such as subjective health and health-promoting or health-risk behaviors) that may help explain the risk of cardiovascular diseases.

Considering that the concepts of well-being and mental health are distinct, it is important to note that adolescent well-being is multidimensional, incorporating both positive aspects (e.g., happiness, engagement) and aspects of ill-being (e.g., depressed mood, feelings of stress) of one’s life (Jarden et al., 2021). Moreover, adolescent well-being is related to different outcomes, such as school engagement, school achievement, life satisfaction, hope, gratitude, physical vitality, and physical activity (Seligman et al., 2009; Kern et al., 2015).

More recent perspectives consider adolescent well-being to be an even broader concept, viewing it as a personal and societal good in its own right: “adolescents have the support, confidence, and resources to thrive in contexts of secure and healthy relationships, realizing their full potential and rights” (Ross et al., 2020, p. 473). The authors propose five interconnected domains that contribute to adolescent well-being and comprise both subjective and objective constructs, including good health, connectedness and contribution to society, education, and agency and resilience.

This conceptual framework for adolescent well-being is consistent with—and underlies—the concept of health-promoting school—“a school that constantly strengthens its capacity as a safe and healthy setting for living, learning and working” (World Health Organization and United Nations Educational, Scientific and Cultural Organization, 2021, p. 1). Despite schools being a privileged setting to improve the health and well-being of students, school-based interventions should also aim to reduce or prevent pathology and problem behaviors (e.g., depression, alcohol use, bullying; American Psychological Association, 2023).

Grounded in this framework, the program #EntreViagenseAprendizagens was developed as a school-based intervention aimed at promoting the overall well-being of adolescents and their positive and healthy development, based on social and emotional learning, health literacy and healthy lifestyles promotion, and positive psychology interventions.

### 1.2. Scientific-based approaches to the promotion of adolescents’ health and well-being

#### 1.2.1. Social and emotional learning

The Social and Emotional Learning (SEL) framework was proposed in 1994 by the Collaborative for Academic, Social, and Emotional Learning (CASEL, 2023), with the aim of establishing SEL as an integral part of education in the school context, based on scientific evidence. School-based SEL interventions involve implementing practices and policies that help students and adults “acquire and apply knowledge, skills, and attitudes to develop healthy identities, manage emotions and achieve personal and collective goals, feel and show empathy for others, establish and maintain supportive relationships, and make responsible and caring decisions” (CASEL, 2020). This occurs through the promotion of a diversity of social and emotional skills, which can be grouped into five major domains, including a variety of thoughts, attitudes, and behaviors: self-awareness (e.g., identifying and understanding one’s emotions), self-management (e.g., identifying and using stress-management strategies), social awareness (e.g., demonstrating empathy and compassion), relationship skills (e.g., communicating effectively), and responsible decision-making (e.g., identifying solutions for personal and social problems).

Different studies have developed meta-analysis of school-based universal interventions and have demonstrated that SEL interventions significantly improved social and emotional skills, attitudes, and behaviors, well-being and academic performance, and reduced emotional and behavioral problems of participants from kindergarten through high school (Durlak et al., 2011; Taylor et al., 2017; Blewitt et al., 2018). Nowadays, SEL interventions are considered a public health approach to education, since they have the potential to improve the general population’s health and well-being (Greenberg et al., 2017). According to CASEL (2020), the integration of SEL into the school’s academic curriculum and the close collaboration with families and the community are beneficial to the effectiveness of SEL programs.

#### 1.2.2. Health literacy and healthy lifestyles promotion

Health literacy is defined as one’s “knowledge, motivation and competences to access, understand, appraise and apply health information in order to make judgements and take decisions in everyday life concerning health care, disease prevention and health promotion to maintain or improve quality of life during the life course” (World Health Organization, 2013, p. 4). It has been found to be associated with healthy behaviors and positive health outcomes in children, adolescents, and adults (e.g., Hsu et al., 2014; Fleary et al., 2018; Svendsen et al., 2020). Therefore, addressing health literacy from an early age is a promising investment in the health and well-being of individuals well into adulthood (Bröder and Carvalho, 2019), and it also has benefits for society, such as economic and social growth (World Health Organization, 2021). Despite its importance for public health, the majority of adolescents from European countries still only have a moderate level of health literacy (Paakkari et al., 2020). Approaches to improving health literacy education in schools are lacking worldwide (Pleasant et al., 2019; World Health Organization, 2021), including in Portugal.

Considering health literacy as a learning outcome in schools, Paakkari and Paakkari (2012) suggested a conceptual model of health literacy which includes two essential components—self-awareness and citizenship—besides the ones that constitute the commonly-accepted concept of health literacy (theoretical knowledge, practical knowledge, and critical thinking). The authors argue that children and adolescents need to understand themselves, others, and the world to make conscious and ethical decisions about health. For this reason, schools have an important role in promoting all these interrelated health literacy components that go beyond basic or functional health literacy (Paakkari and Paakkari, 2012).

In addition to delivering factual or practical information about health or healthy lifestyles, it is important to promote critical reflection and personal meaning-making processes among the students (Paakkari and Paakkari, 2012). For this reason, intervention strategies related to health literacy and healthy lifestyles should consider the adolescents’ daily life, include hands-on and practical activities (Bröder and Carvalho, 2019), involve parents/caregivers (Pleasant et al., 2019), take a holistic approach, and target multiple behavioral changes simultaneously (Irvine et al., 2022). Considering the general trend of decreasing healthy lifestyles during adolescence, interventions must especially focus on improving physical activity and healthy eating, and on reducing screen time and substance use, which may also be beneficial for concurrent reductions in symptoms of depression and anxiety (e.g., Gunnell et al., 2016).

Given the recent increase in mental health problems among young people around the world, following the COVID-19 pandemic (Deng et al., 2023), special focus on mental health literacy has become increasingly necessary, since it is considered a prerequisite for early recognition, management, prevention, and intervention in mental disorders (Jorm, 2000), but also a necessary skill to maintain and obtain a good mental health (Kutcher et al., 2016). School-based interventions should promote positive mental health, but also help students “to differentiate normal mental distress from mental health problems/disorders, reduce stigma against mental illness, and promote help-seeking behaviors of students and mental health self-care if they need mental health care” (Kutcher et al., 2016, p. 568). Although the interventions specifically aimed at promoting mental health literacy in schools are few, there have been some positive results from short interventions that resulted in improvements in knowledge and use of self-help strategies and first-aid skills, as well as decreased stereotyping associated with increased knowledge about mental health problems (e.g., Skre et al., 2013; Campos et al., 2018). Additionally, “teen Mental Health First Aid” programs have also been found to be effective in improving mental health literacy, confidence in providing mental health first aid to peers, help-seeking intentions, and student’s mental health, as well as in reducing stigmatizing attitudes (e.g., Hart et al., 2016).

#### 1.2.3. Positive psychology interventions

Current perspectives in Positive Psychology consider a focus on positive life trajectories to be highly important for the promotion of strengths, well-being, and other positive outcomes, beyond the reduction of negative outcomes, in particular among children and adolescents (Norrish and Vella-Brodrick, 2009; Owens and Waters, 2020). Furthermore, this focus can contribute to positive human functioning and to individual, interpersonal, and societal flourishing (Seligman and Csikszentmihalyi, 2000). For this reason, Seligman et al. (2009) consider that positive education—i.e., “an adaptation of traditional forms of education focused on building academic competencies, blending the knowledge of well-being science with effective pedagogy to promote learning for traditional academic skills, optimal development, and wellbeing” (Oades et al., 2021, p. 293)—should be implemented in all schools. Well-being literacy (i.e., the capacity to understand and intentionally use well-being concepts or components to maintain or improve the well-being of oneself or others, taking into account the specific context) is, nowadays, considered a key competence underlying positive education pedagogy (Oades et al., 2021).

Character strengths interventions have proliferated since the publication of the empirically-driven classification of character strengths and virtues by Peterson and Seligman (2004), where these strengths are identified as relevant factors for promoting well-being and buffering against psychological disorders among youth. This system (Values-In-Action Strengths Classification) is composed of 24 ubiquitous character strengths (positive traits reflected in thoughts, feelings, and behaviors), organized into six broad virtues—courage, wisdom and knowledge, temperance, justice, humanity, and transcendence. Various character strengths-based interventions for adolescents, which focused on recognizing and exercising “signature strengths” in daily life, proved to have an impact on the life satisfaction, well-being, and flourishing of participants (e.g., “Strengths Gym,” Proctor et al., 2011). Considering that a more frequent use of character strengths is associated with life purpose (Kashdan et al., 2022), these constructs should be jointly addressed with adolescents. Research has also shown that interventions focused on life purpose or meaning are scarce (e.g., Burrow et al., 2022), but they seems to contribute to the improvement of adolescent well-being, resilience, and physical and mental health (Steger et al., 2021).

Other school-based positive psychology interventions with adolescents, focused on resilience, gratitude, kindness, or positive emotions, have led to improvements in well-being, pro-social behavior, and school performance, but also to a reduction in psychopathological symptoms (Norrish and Vella-Brodrick, 2009; Owens and Waters, 2020). For example, RESCUR, a universal curriculum that promotes resilience in children and adolescents from schools of six European countries, has shown very positive results in decreasing mental health difficulties and increasing both pro-social behaviors and well-being (e.g., Cefai et al., 2014; Simões et al., 2021). Gratitude building interventions developed with children and adolescents have also shown positive effects on psychological well-being, positive affect, positive feelings, life satisfaction, and gratitude (e.g., Froh et al., 2014; Khanna and Singh, 2016). Layous et al. (2012) developed a four-week intervention specifically to encourage preadolescents to perform acts of kindness and found improvements in the students’ well-being and peer acceptance.

### 1.3. The present study protocol

The aim of the present study protocol is to describe the intervention and evaluation protocol of #EntreViagenseAprendizagens, a multi-component school-based intervention developed on the basis of the different approaches mentioned above (i.e., social and emotional learning, health literacy and healthy lifestyles promotion, and positive psychology), with a view to promoting well-being and healthy lifestyles among adolescents.

## 2. Methods and analysis

### 2.1. Selection of participants

Students in the 8th grade from three public schools of the Lisbon metropolitan area will be invited to participate in #EntreViagenseAprendizagens. The criteria for participation in the program include being enrolled in the 8th grade, agreeing to participate in the study (as part of the intervention or the control group), and having their parents or guardian’s formal authorization to participate. All participants will read and sign an informed consent form, which states the objectives of the study and intervention and ensures the confidentiality of the data provided in the questionnaire responses (and of the content of the reflections shared in the sessions, in the case of intervention group participants).

### 2.2. Intervention

#EntreViagenseAprendizagens is a 20-week school-based intervention that uses the metaphor of a journey to a better world, for which students must prepare through learnings and skills they put in their “luggage.” These learnings and skills are obtained by “making a stop” at different places in the city (e.g., “Market of Emotions,” “Mind Gym”), which correspond to the various sessions. This metaphor aims to illustrate the preventive and holistic nature of the intervention.

As recommended for effective SEL programs, the design of the program was based on the SAFE (acronym for Sequenced, Active, Focused, and Explicit) approach (Durlak et al., 2011). Indeed, #EntreViagenseAprendizagens adopts a sequential training approach (e.g., activities on emotion recognition precede activities on emotion regulation strategies), uses active forms of learning, based on experiential and participatory activities (e.g., role-playing, debates), devotes sufficient time to skill development, and has explicit learning goals. The development of skills is facilitated by experiential and participatory learning during the sessions, but their application in other contexts is also promoted, notably through challenges proposed to be carried out during the week (e.g., activities to be developed by the students together with their families).

**Figure 1:**
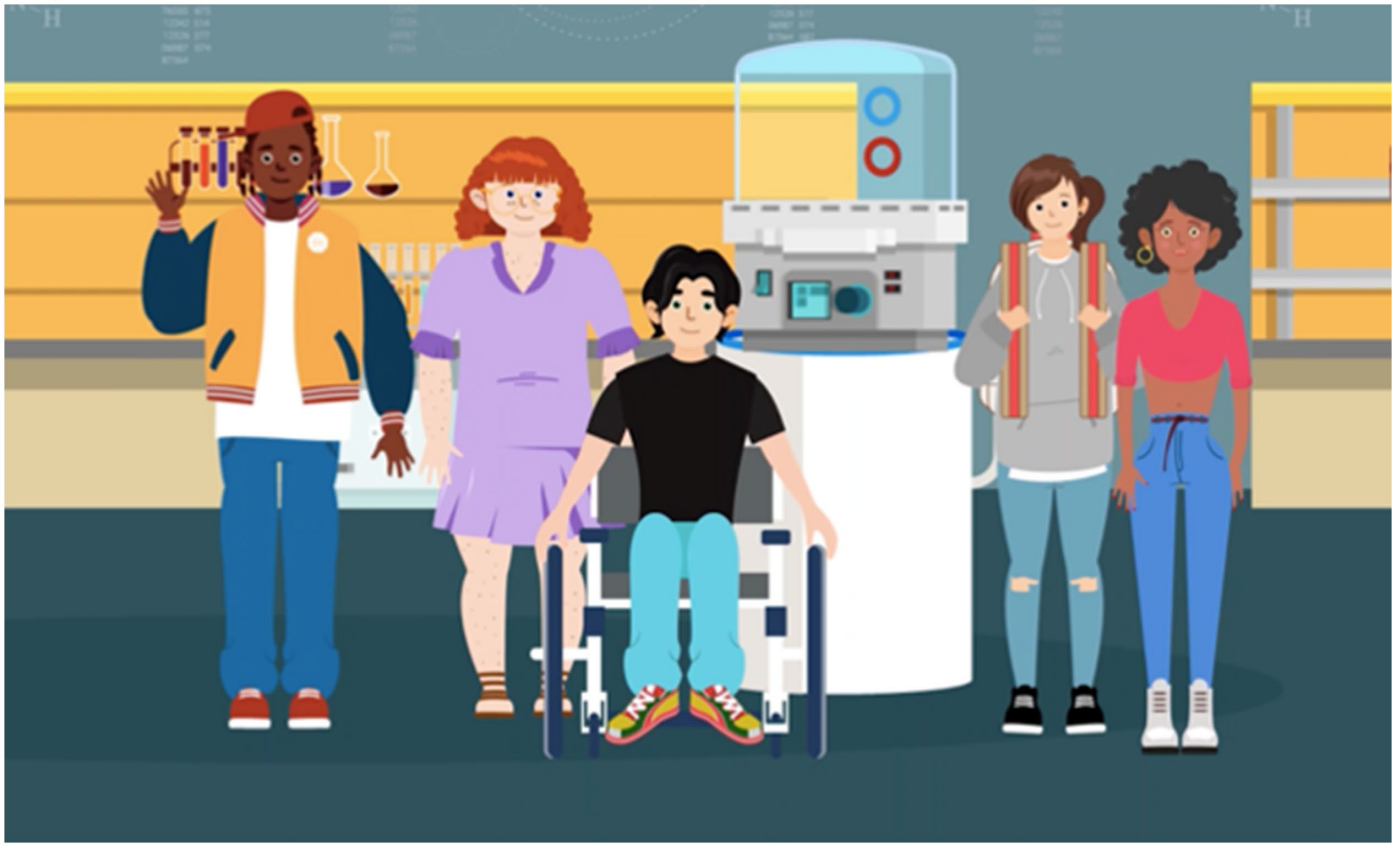
Program characters.

Five characters were created within the scope of the program and will be used to accompany the students throughout the intervention (Figure 1). The characters are teenagers with diverse characteristics (e.g., characters with different ethnicities and weights, a character with impaired mobility). The aim is to make it easier for all the students to identify with the themes of the program and to enhance their engagement with the intervention. The characters are featured in the four videos designed specifically for this program, which focus on themes covered in the sessions (#HealthLiteracy, #MentalHealthLiteracy, #CharacterStrengths, #Communication) and in the activity sheets used during the program. An Instagram^®^ account for #EntreViagenseAprendizagens will be set up and used throughout the program to recall the “healthy tips” and run the “Well-being-Promoting Actions” photo contest (see below).

A pilot version of the program, including 10 sessions of 90 min each (Francisco et al., 2019b) and involving 54 students from 2 schools, was implemented and evaluated. The results of the pilot study revealed statistically significant improvements in some aspects of the participants’ lifestyle (more hours of sleep on the weekend, increased soup consumption), in their emotional clarity and in two dimensions of their well-being (connectedness and optimism). Participants also reported a high level of satisfaction with the program. The qualitative data revealed that the main learnings and changes identified by the participants relate to improvements in self-awareness, emotion regulation skills, and healthy eating. The most valued aspects of the program regarding its procedures and activities were the debates and discussions, the active role-playing skills training, and the dynamic relationship created between all the participants, including the program facilitators. The participants suggested that a future version of the program should address mental health issues (Hormigo and Francisco, 2019). These findings led to some modifications, such as the inclusion of sessions specifically dedicated to mental health literacy, and a session with the participants’ parents, considering the importance of family to the expected outcomes. Additionally, the duration of the sessions was reduced (and therefore the total number of sessions increased) to make it easier to integrate the program into the school curriculum (which in Portugal includes 50-min lessons).

#### 2.2.1. Dimensions, themes, and skills covered by #EntreViagenseAprendizagens

Considering the five domains of social and emotional skills, proposed by CASEL (2020), some specific skills were selected from each domain, based on their relevance to the age of the target population and their relationship with overall well-being, as shown in the literature and explained above. Thus, the program essentially focuses on the skills of emotion recognition, emotion regulation, self-control, self-esteem, empathy, communication, cooperation, and problem-solving, particularly in nine of its sessions. However, certain skills, especially those related to social awareness and relationships, are worked on transversally, since most of the proposed activities are developed in groups (e.g., debates, joint reflections). In turn, the video #Communication, used in the session especially dedicated to assertive communication, addresses a health-related theme—adolescent smoking—and its relationship with responsible decision-making and communication with parents, which illustrates the interrelation and integration of the different approaches, oriented towards the promotion of the overall well-being of the participants.

Six sessions were specifically created to promote health literacy and healthy lifestyles. Besides one session focused on general health literacy, which uses the video #HealthLiteracy to introduce the theme, three other sessions were designed to address different aspects of healthy lifestyles that are particularly critical for adolescents, such as eating habits and physical activity, but also screen use, sleep, and tobacco and alcohol consumption. In addition, the video #MentalHealthLiteracy introduces two specific sessions focused on the identification of different mental health problems (e.g., depression, anxiety, eating disorders), first-aid skills, and help-seeking strategies. Whitin the realm of prevention strategies and promotion of well-being and mental health, a session was specifically designed to focus on relaxation and mindfulness exercises, which are widely employed in positive psychology interventions.

Other themes related to positive psychology interventions are the subject of five sessions in particular. A special focus is placed on the theme of gratitude and empathy, highlighting the importance of positive interpersonal relationships and social connectedness for subjective well-being. The theme of subjective well-being is also present throughout the program, through the photo contest “Well-being-Promoting Actions,” as will be explained below. Finally, the video #CharacterStrengths introduces the theme referred to in its title during a session designed to help students to find their character strengths (Peterson and Seligman, 2004). Their “signature strengths” are then used to work on themes such as purpose in life, commitment, adaptability, and resilience.

Table 1 presents the summary of the main skills to be developed throughout the 20 sessions (19 sessions for adolescents and one session for parents) and the main activities for each session.

**Table 1:** Summary of the content of the sessions.

Session	Main skills to be developed	Main activities
#01 “Zero Street”	Subjective well-being	-Icebreaking with students: name one thing you like and another you do not like -Presentation of the program -Debate about “a better world” and suggestions for improving today’s world (e.g., What is the world like now? What would be the perfect world? What solutions exist? How can everyone contribute?) -Individual reflection on situations that contribute to well-being and for which each student is grateful
#02 “Health Roundabout”	Health literacy	-Debate about health (e.g., What does health mean to you? What makes you sick? What do you do to stay healthy?) and reliable information sources -Viewing of the video #HealthLiteracy -Discussion about the video: “What would you do in Maria’s situation?” -Reflection on the concept of overall health
#03 “Market of Emotions”	Emotion recognition	-“EmoImage”: identify the five basic emotions in images and classify them (sadness, anger, joy, fear, and disgust) -“Where do you feel emotions?”: drawing a body and painting where emotions are felt -Reflection on students’ perception of emotions and the differences and similarities between the paintings of the various groups
#04 “All Aboard Bus” [for parents]	Emotional literacy Communication Relational well-being	-“Verbal and Non-Verbal Communication Act”: debate about communication styles -“Charades”: discussion about effective communication with adolescents -“Emotional Awareness: Situations Sample”: promotion of emotional awareness -“Action skills”: sharing emotional regulation strategies -Reflection on how relationships promote well-being
#05 “Pharmacy of Feelings”	Emotion recognition Self-regulation	-“Thermometer of emotions”: identify situations (related to school, family, friends, and individual) and paint the thermometer according to the intensity of the emotion -Reflection on emotional regulation strategies that could be implemented in the situations identified by the students (e.g., “how can I identify and reduce triggers?”; I should take a breath and slow down the moment between trigger and response; notice what I feel; name the emotion; accept it; engage in positive self-talk and/or make a choice about how to respond)
#06 “Solutions street”	Self-regulation Problem-solving	-Emotional Regulation Activity: discussion about how to adopt attitudes and behaviors adjusted to different emotionally intense situations typically experienced by adolescents -“Let us find out”: debate on the set of solutions for the different situations presented
#07 “*EntreViagens* Restaurant”	Health literacy: Healthy eating	-Discussion about “What is a Healthy Diet?” -Video about the Food Wheel and educational presentation about the principles of the Mediterranean Diet -“Let us count sugar packets!”: some foods are presented to the students (e.g., iced tea, chocolate milk, white milk, apple, cereal bar) and they have to find out how many packets of sugar are contained in each one -“Label Decoder”: reading the labels of some food items
#08 “Mind Gym”	Health literacy: Physical activity	-“Who is who?”: discussion about the differences between physical activity, exercise, sport and sedentary behavior -“Pyramid of time”: presentation of the physical activity pyramid and discussion about the minimum time of daily physical activity, aerobic exercise, and strength and flexibility training -“Let us go practice”: Four practical exercises (e.g., squats, jumping jacks)
#09 “Garden of Balance”	Health literacy: Healthy lifestyles	-“Quiz time”: group quiz about healthy lifestyles (screen use, sleep, diet, physical activity, and alcohol, tobacco, and drug use) -Discussion about the key ideas
#10 “Mirror Shop”	Character strengths Adaptability	-Viewing and discussion of the video #CharacterStrengths -“Find out who you are”: filling out the *VIA* Survey of Character Strengths -Debate about individual differences between participants and examples of how to use their “signature strengths”
#11 “Central square”	Mental health literacy Empathy	-“Let us talk about mental health problems”: identification of symptoms and ways of helping someone who is experiencing a mental health problem (depression, anxiety or anorexia nervosa) -Viewing and discussion of the video #MentalHealthLiteracy
#12 “School of the mind”	Mental health literacy Self-regulation	-Identification of and group reflection on anxiety-producing situations in the school context -Discussion about different behaviors/strategies that can be used to prevent or deal with anxiety in those situations
#13 “Connection Club”	Self-regulation Subjective well-being	-“Time to relax…”: guided practice of mindfulness and progressive muscle relaxation exercises -Reflection on and discussion about the impact of both exercises on the participants’ body and mind
#14 “Parliament”	Communication	-Viewing and discussion of the video #Comunication -Reflection on the different styles of communication (aggressive, passive, and assertive) -“Let us communicate!”: role-play of the different styles of communication
#15 “The Empathy Clinic”	Empathy Communication Subjective well-being	-Presentation of a video about empathy -Joint reflection on and discussion about the meaning of “empathy” and its impact on personal and social well-being -“Let us practice empathy!”: role-play of different situations representing empathetic attitudes and behaviors
#16 “Garden of the future”	Ecological Literacy Communication Problem-solving	-Presentation of a video about climate change -Joint reflection on and discussion about the challenges facing the planet -“Let us find solutions for our planet!”: debate about measures that contribute to environmental sustainability vs. difficulties in implementing these measures
#17 “Surprise!”	[Theme chosen by parents]	[To be defined according to the chosen theme]
#18 “Filter Factory”	Self-esteem Self-regulation	-Presentation of a video about self-esteem and social media filters -Joint reflection on and discussion about the impact of social media on adolescents’ self-esteem -Debate about the pros and cons of the use of social media
#19 “Avenue of Well-Being”	Life Purpose Commitment Resilience	-Sharing and debate about the participants’ Vision Board (images that represent what each participant wants to be, have, or achieve in the future) previously created at home -Reflection on how their “signature strengths” are a fundamental resource for achieving their goals for the future
#20 “Boarding gate”	End of program	-A tour of the program -Certificates of participation and photo contest awards

#### 2.2.2. Goals and hypotheses

The main goal of #EntreViagenseAprendizagens is to promote well-being and healthy lifestyles among adolescents. Specifically, the program aims to: (a) foster social and emotional skills (particularly self-regulation, communication, problem-solving, resilience, and adaptability); (b) identify and strengthen the students’ virtues and character strengths, which contribute to the definition of their life purpose and to their ability to adapt; (c) increase general health literacy and mental health literacy; (d) improve knowledge and behaviors related to healthy lifestyles, especially physical activity and healthy eating; and (e) improve overall well-being. Indirectly, the program also serves the purpose of preventing the development of social and emotional adjustment problems (e.g., anxiety, depression, behavioral problems).

We hypothesize that the #EntreViagenseAprendizagens program will result in (1) a significant increase in social and emotional skills (self-control, cooperation, empathy, and stress resistance); (2) a significant decrease in emotion regulation difficulties (specifically, limited access to emotion regulation strategies and lack of emotional clarity); (3) a significant increase in general health literacy and mental health literacy (specifically, self-help strategies, first-aid skills and help-seeking, and knowledge about mental disorders); (4) a significant increase in knowledge about nutrition and physical activity; (5) a significant increase in health-related attitudes and behaviors (e.g., increase in physical activity and healthy eating, decrease in screen time); (6) a significant improvement in the perception of the quality of relationships with others (specifically, peer acceptance and relationships with mother and father); and (7) a significant increase in adolescent well-being (specifically, engagement, perseverance, optimism, connectedness, and happiness). We also hypothesize that the results will be maintained at the 9-month follow-up.

#### 2.2.3. Structure of sessions with students

The sessions are implemented with groups of 12–15 students, with one facilitator per group. All sessions have clearly defined learning goals, and are dynamic and interactive, with a duration of 50 min (i.e., the same duration as regular curricular classes, in order to maximize the adolescents’ attention and encourage adoption of the program by creating the possibility of integrating it into regular curricular units). Most sessions are structured as follows: a semi-structured individual (or group) activity, a semi-structured activity in small groups, and a final interactive debate on what was learned during the session.

Although there are always one or two key skills that are the focus of each session, in the same session several competencies can be worked on, given their association with different themes and the use of different types of activities. For example, in session #14, titled “Parliament,” the main theme is communication (focusing on assertive communication as an important social and emotional skill); however, the themes covered in the scenarios presented to the students for the role-play concern different issues, such as (un)healthy behaviors, making it possible to work on health literacy at the same time.

At the end of each session, a “healthy tip” associated with the topic that was addressed during that session (e.g., #gratitude) is discussed and complemented with a “challenge of the week.” This challenge is an intersession activity to be developed during the week (e.g., “gratitude agenda”) and related to the content of the session. This activity can be shared and discussed at the next session if the participants so wish.

In the first session, each student will receive a folder in which to place all the activity sheets completed during the program. This folder will then serve as a portfolio and a record of the student’s evolution and involvement.

#### 2.2.4. Involvement of students’ parents

Before the program begins, all parents/guardians of students in the intervention group will be invited to attend an online session, where the objectives of the program will be presented and doubts will be clarified. This session aims to promote family involvement from the beginning of the program and to motivate families to participate in some of the challenges of the week. As defined by the American Psychological Association (2023), school-based interventions should include special homework assignments to be completed with parents. Therefore, alerting parents to its relevance is essential from the start.

The fourth session of the program is aimed at the parents/guardians, focusing on their role in promoting the well-being of the students. In this session, the parents will also be asked to vote on a topic they consider relevant to be subsequently included in the “surprise” session of the program (session 17). Examples of such topics are bullying and peer pressure.

#### 2.2.5. Photo contest “well-being-promoting actions”

The program includes a photography competition, titled “Well-being-Promoting Actions,” associated with the photovoice methodology (Wang and Burris, 1997) and run on the program’s Instagram® account. The contest aims to promote the involvement of the students, their families, the school community, and the community in general as well as amplify the effect of the intervention. The use of photovoice will allow students to engage their creativity and become more interested in reflecting and writing about their well-being, increasing their well-being literacy (Oades et al., 2021). It will also contribute to improving their self-esteem and self-determination, as the students feel respected and considered (Golden, 2020).

Students will take a photo of something they do that they think contributes to their well-being and write a five-line paragraph explaining why they chose it and what the photo says about their perception of health and well-being. The photos will be posted on the Instagram^®^ page of #EntreViagenseAprendizagens, where the voting will take place. In each school, the three best photographs (with the most “likes”) will be elected, and their authors will receive a prize (e.g., book/music shop vouchers). The competition may result in a photo exhibition at the schools, at the end of the school year. The aim of the exhibition is to integrate the learnings acquired during the program and showcase them to the rest of the school, thus promoting a sense of belonging and normalization of the themes addressed. This exhibition may also help the whole school community to reflect on these themes.

#### 2.2.6. Context of implementation and facilitators

The program can be implemented in two different formats: (a) integrated into an 8th or 9th grade subject at the choice of each school (e.g., Citizenship and Development), running for two periods of the school year; (b) as an extracurricular activity of the schools. The first option allows for a greater number of participants and a broader scope of the program. In the evaluation study of #EntreViagenseAprendizagens, only students from the 8th grade attending the regular curriculum (subject of Citizenship and Development) will be included.

The program will be facilitated by psychologists with a master’s degree in Psychology, preferably with experience in therapeutic intervention with adolescents. The facilitators will receive training from the first author (project coordinator) regarding the #EntreViagenseAprendizagens program, which will cover, among other aspects, the objectives and contents of each session. Biweekly follow-up meetings with the project coordinator will be held to deal with any unforeseen circumstances that may arise, highlight important aspects of each session, deliver materials, monitor the adequacy and fidelity of the implementation, and brainstorm about aspects that might be improved.

### 2.3. Intervention impact evaluation

#### 2.3.1. Design and procedure

A cluster randomized controlled trial design will be used for impact evaluation, with 8th grade classes (units of randomization) from three public schools being assigned to either the intervention or the control condition. Data collection will be based on structured questionnaires applied to all students, both in the control group and the intervention group. Baseline measures (T0) will be collected with all potential participants prior to intervention implementation. In each school, three classes will then be randomly selected to be part of the intervention group in Year 1, while the remaining classes will be the control group. Students who integrate the control group in Year 1 will be part of the Intervention Group in Year 2. The post-test (T1) will take place approximately 1 week after the end of the program and the follow-up (T2) will take place 9 months later.

#### 2.3.2. Instruments

##### 2.3.2.1. Social and emotional skills

Social and emotional skills will be assessed using two instruments.

Four subscales (with eight items each) of the Organization for Economic Co-operation and Development (OECD) Study on Social and Emotional Skills (SSES; Organisation for Economic Co-operation and Development, 2021) will be used to evaluate self-control (e.g., “I stop to think before acting”), cooperation (e.g., “I am always willing to help classmates”), empathy (e.g., “I understand what others want”), and stress resistance (e.g., “I am relaxed and handle stress well”). The items are answered on a 5-point Likert-type scale from 1 (completely disagree) to 5 (completely agree). The Portuguese version that will be used presents satisfactory levels of internal consistency (ranging from *α* = 0.67 to *α* = 0.74 for the subscales that will be used; Organisation for Economic Co-operation and Development, 2021).

Two subscales of Difficulties in Emotion Regulation Scale (DERS; Gratz and Roemer, 2004) will be used to assess two components of emotion regulation, specifically “limited access to emotion regulation strategies” (eight items; e.g., “When I’m upset, it takes me a long time to feel better”) and “lack of emotional clarity” (five items; e.g., “I am confused about how I feel”). These items are answered on a 5-point Likert-type scale from 1 (“almost never applies to me”) to 5 (“almost always applies to me”). The higher the score on each of the subscales, the greater the participants’ emotion regulation difficulties. The Portuguese version that will be used presents good internal consistency (*α* = 0.88 and *α* = 0.75, respectively) for these two subscales (Coutinho et al., 2010).

##### 2.3.2.2. Well-being

The EPOCH Measure of Adolescent Well-Being (Kern et al., 2016) will be applied to evaluate five positive psychological characteristics considered to contribute to well-being, physical health, and other positive outcomes: engagement (e.g., “I get completely absorbed in what I am doing”), perseverance (e.g., “I finish whatever I begin”), optimism (e.g., “I am optimistic about my future”), connectedness (e.g., “When I have a problem, I have someone who will be there for me”) and happiness (e.g., “I feel happy”). Each subscale is composed of four items, answered on a 5-point Likert-type scale from 1 (almost never/not at all like me) to 5 (almost always/very much like me). Both the original and the Portuguese version that will be used (Francisco et al., 2019a) present good internal consistency (from *α* = 0.74 to *α* = 0.86, and from *α* = 0.82 to *α* = 0.93, respectively).

##### 2.3.2.3. Health literacy

The Health Literacy for School-Aged Children (HLSAC; Paakkari et al., 2016) will be used to assess students’ subjective health literacy. It is composed of 10 items, two items from each of five predetermined theoretical components: theoretical knowledge (e.g., “I have good information about health”), practical knowledge (e.g., “When necessary, I find health-related information that is easy for me to understand”), critical thinking (e.g., “I can usually figure out if some health-related information is right or wrong”), self-awareness (e.g., “I can give reasons for choices I make regarding my health”), and citizenship (e.g., “I can judge how my own actions affect the surrounding natural environment”). The items have a 4-point Likert-type response scale from 1 (not at all true) to 4 (absolutely true). The sum of the answers allows the identification of the participants’ levels of health literacy: “low” (score 10–25), “moderate” (score 26–35), and “high” (score 36–40; Paakkari et al., 2018). Both the original (Paakkari et al., 2016) and the Portuguese version (Francisco, 2020) that will be used present good internal consistency for total score (*α* = 0.93 and *α* = 0.87, respectively).

Twelve items from the young people version of the Mental Health Literacy Questionnaire (MHLq; Campos et al., 2016), directly related to the contents on mental health literacy covered by #EntreViagenseAprendizagens, were selected to serve as indicators of self-help strategies (four items; e.g., “Physical exercise helps to improve mental health”), first aid skills and help-seeking (four items; e.g., “If a friend of mine developed a mental disorder, I would encourage her/him to get medical support”), and knowledge/stereotypes (four items; e.g., “Mental disorders affect people’s thoughts”) about mental health. The items have a 5-point Likert-type response scale from 1 (strongly disagree) to 5 (strongly agree). The original version of this questionnaire, with 34 items, presents good internal consistency (from *α* = 0.72 to *α* = 0.79 for each factor, and *α* = 0.84 total score).

##### 2.3.2.4. Health-related knowledge, attitudes, and behaviors

To evaluate knowledge, attitudes, and behaviors associated with health, 14 items answered on a Likert-type scale from HBSC studies (Inchley et al., 2018) will be used. They relate to physical activity (e.g., “In the last 7 days, in how many days did you accumulate at least 60 min of physical activity (e.g., gymnastics, sports, playing football, walking to school, etc.)?”), alcohol and tobacco consumption (e.g., “How often do you smoke tobacco?”), screen time (“In your free time, during the week, how much time per day do you use screens such as iPads, television, cell phones, or computers?”), eating habits (e.g., “During the week how often do you eat soup?”), and sleep habits (e.g., “How many hours, on average, do you sleep at night on weekdays?”).

Five multiple-choice questions, adapted from the Questionnaire of Nutrition (NUT-Q; Raich et al., 2008), will be also used to measure knowledge of nutrition and particular types of food. For example: “Which of the following nutrients constitute the body’s main energy reserve?” (possible answers: proteins; vitamins and minerals; fats; carbohydrates; I do not know). The sum of the correct answers corresponds to the total value for this dimension (ranging from 0 to 5).

Four items, taken from the Portuguese barometer for physical activity (Silva et al., 2018), will be used to assess students’ knowledge about physical activity. Participants indicate on a 5-point Likert-type scale their level of agreement with the sentences presented (e.g., “Climbing stairs or walking is not physical activity,” “Only high-intensity physical activity has beneficial effects”).

##### 2.3.2.5. Relationships with others

Specific items from HBSC studies (Inchley et al., 2018) will be used to evaluate students’ peer acceptance (e.g., “My classmates accept me for who I am”) with a Likert-type scale from 1 (“False most of the times”) to 3 (“True most of the times”), as well as their participation in situations of conflict or violence (e.g., “How many times have you taken part in provocations to another student(s), in the last 2 months?”), with a five Likert-type scale from 1 (“I did not take part in provocations”) to 5 (“Several times a week”).

Two items to assess the students’ relationship with both parents will be also presented (e.g., “How do you evaluate your relationship with your mother?”), answered on a 5-point Likert-type scale from 1 (“Very bad”) to 5 (“Very good”).

##### 2.3.2.6. Sociodemographic data

Information about the sociodemographic characteristics of students and their parents will be collected for the present study, including sex, age, special educational needs, household, and parents’ marital status and level of education, among others.

#### 2.3.3 Data analysis

Descriptive statistics will be used to describe intervention and control group participants, replying to the pre-test, post-test, and follow-up questionnaires. To assess intervention effectiveness, statistically significant differences (and effect sizes) between the intervention and control groups will be examined by repeated measures ANOVA, contrasting results from the intervention and control groups (between-subjects factor) on the different outcome measures (e.g., social and emotional skills, health literacy, well-being) at pre-test, post-test, and follow-up (within-subjects factor).

### 2.4. Intervention process evaluation

#### 2.4.1. Design and procedure

Process evaluation will rely on a mixed-methods approach, combining quantitative (i.e., questionnaires) and qualitative methods (e.g., focus groups). Students in the intervention group will participate in an initial qualitative assessment of their expectations and a final global assessment, in terms of their satisfaction with the intervention and intervention quality, using a questionnaire, with both closed questions (Likert-type scales) and open-ended questions. The program facilitators will also complete a session evaluation sheet at the end of each session, in order to check intervention fidelity. Additionally, sessions and program evaluation sheets will be applied immediately after each session to participants of the intervention group. At the end of the program, focus groups will be held with: (1) students from the three schools where the program will be implemented (one per intervention group); (2) three focus groups with parents (one per school); and (3) one focus group with teachers from the subject into which the program will be integrated and/or the class director (i.e., the teacher who is responsible for a particular class in school).

#### 2.4.2. Instruments

##### 2.4.2.1. Session evaluation sheets

Session evaluation sheets will be filled in after each session, both by intervention participants and facilitators. The students’ post-session evaluation questionnaires will include: (1) one item evaluating overall satisfaction with the session (i.e., “In general, did you like today’s session?”), answered on a Likert-type scale, ranging from 1 (“Did not like it”) to 4 (“Liked it very much”); (2) five items assessing the session’s perceived relevance (“Was the session important?”), interest (“Was the session interesting?”), and challenge (“Was the session challenging?”), as well as the degree to which the students feel they have developed their competences (“Do you feel that you have developed your skills?”) and whether they had difficulty in concentrating (“Did you feel difficulty in concentrating?”), all answered on a Likert-type scale ranging from 1 (“Not at all”) to 4 (“Very much”); and (3) two open-ended questions, where participants can mention the most and least appreciated aspects of the session (e.g., “What did you like the most in today’s session?”).

The sessions’ evaluation sheets completed by the facilitators to register relevant data at the end of each session (e.g., participants’ attendance, themes/contents covered, any deviations from the plan for each session) will be used to check intervention fidelity (i.e., whether the program is being implemented as planned). Their content will be discussed during the biweekly follow-up meetings with the project coordinator.

##### 2.4.2.2. Overall program evaluation questionnaire

At the end of the intervention, an overall intervention evaluation questionnaire will be applied to participants, including: (1) one item to collect a global assessment of the intervention, answered on a Likert-type scale ranging from 1 (“Did not like it”) to 4 (“Liked it very much”); (2) seven open-ended questions, with the purpose of identifying the program features and components that pleased the intervention participants the most and the least, what they consider to have learned, and their suggestions regarding changes that could be applied in future program implementations.

##### 2.4.2.3. Focus group interview guides

Semi-structured interview guides for the focus groups with participants, parents, and teachers will be structured around four main topics: (1) whether they consider the program to be beneficial; (2) what sessions, themes, activities, and components they (or their children/students) liked the most and the least, and why; (3) what they (or their children/ students) consider to have learned, and whether they perceived any changes in relation to the social and emotional skills targeted by the program (e.g., emotion regulation, communication), well-being, relationships with others, health literacy, and/or lifestyles; (4) suggestions regarding changes for future versions of the program (e.g., new contents and implementation logistics, such as scheduling, sessions duration and dynamics, etc.).

The interview guide for parents will also include questions regarding the usefulness of the program session dedicated to parents and their experiences with the activities that required the parents’ involvement (e.g., “challenges of the week” activities). The interview guide for teachers will also include questions regarding the adequacy of the integration of the program into a regular curricular subject (e.g., Citizenship and Development) and alternative forms of program implementation (e.g., as extracurricular activity).

#### 2.4.3. Data analysis

Descriptive statistics will be used to describe the evaluation of each session and of the overall program made by the participants. To determine whether specific sessions are perceived as being significantly more/less interesting, challenging, and relevant, whether participants perceived the session as contributing more/less to the development of new competencies, and whether they felt more/less difficulty in concentrating, analyses of variance (ANOVAs) will be conducted. Thematic analysis (Braun and Clarke, 2006) will be performed on all qualitative data gathered from the session evaluation sheets, overall program evaluation questionnaire, and focus groups transcriptions.

## 3. Dissemination

The plan for disseminating the #EntreViagenseAprendizagens program includes the following measures: (a) publish the results on the effectiveness of the intervention program in peer-reviewed journals; (b) write a technical manual (including all videos and activity sheets to be used) to ensure that the program is implemented in the same way by different facilitators and with different groups; and (c) train program facilitators, through workshops on the theoretical rationale and methods for the implementation of #EntreViagenseAprendizagens in schools, targeting teachers, psychologists and other school and mental health professionals who want to implement this program with adolescents.

## 4. Discussion

The aim of the present paper is two-fold: to describe the content of a school-based intervention designed to promote adolescent well-being through the development of social and emotional skills, health literacy, and healthy lifestyles in 8th and 9th grade students; and to present its evaluation protocol. Despite the numerous school-based interventions focused on social and emotional skills, mental health, and healthy lifestyles, or even specific school-based positive psychology interventions, there have been few interventions aimed at promoting the well-being of adolescents in such a comprehensive way, considering all its dimensions. #EntreViagenseAprendizagens is innovative, since it is a multi-component school-based intervention that takes into account the adolescents’ overall well-being, including their mental and physical health and well-being, alongside character strengths and social and emotional skills, all of which are essential to their future adaptation in a continuously changing world. This is a relevant fact, considering that multi-component interventions are in line with the most recent conceptualizations of adolescent well-being (Ross et al., 2020) and of health-promoting schools (World Health Organization and United Nations Educational, Scientific and Cultural Organization, 2021).

The development of the program is based on solid intervention approaches that have shown promising results, such as social and emotional learning (CASEL, 2020), health literacy promotion (Bröder and Carvalho, 2019), and positive psychology interventions (Seligman et al., 2009). Moreover, the use of an experimental design with pre- and post-intervention measurements for impact evaluation will make it possible to provide solid evidence on the effectiveness of the intervention. If the expected results are achieved, future research should continue to investigate this intervention and its mechanisms for action by standardizing its design. Furthermore, process evaluation based on the use of mixed methods will provide insights into contextual factors and mechanisms that may impact the overall intervention effects, informing future program adaptations, if needed.

It is urgent to invest in a whole-school approach, with the integration of #EntreViagenseAprendizagens and other similar programs into the compulsory school curriculum, which can be adapted to the specific needs of each group of students and allow for the development of real health-promoting schools (World Health Organization and United Nations Educational, Scientific and Cultural Organization, 2021).

## Data availability statement

The original contributions presented in the study are included in the article, and further inquiries can be directed to the corresponding author.

## Author contributions

RF and CG designed the study protocol. RF conceptualized the school-based intervention #EntreViagenseAprendizagens, coordinated the study, and was responsible for funding acquisition. RF, BR, MS, and MH developed the pilot version of #EntreViagenseAprendizagens. AC and AJ contributed to the optimization of the present version of #EntreViagenseAprendizagens. All authors contributed to the article and approved the submitted version.

## Funding

This research is funded by Gulbenkian Academies for Knowledge (Ref. 240626), Calouste Gulbenkian Foundation.

## Conflict of interest

The authors declare that the research was conducted in the absence of any commercial or financial relationships that could be construed as a potential conflict of interest.

## Publisher’s note

All claims expressed in this article are solely those of the authors and do not necessarily represent those of their affiliated organizations, or those of the publisher, the editors and the reviewers. Any product that may be evaluated in this article, or claim that may be made by its manufacturer, is not guaranteed or endorsed by the publisher

## References

[ref1] American Psychological Association . (2023). School-based intervention. APA Dictionary of Psychology. Available at: https://dictionary.apa.org/school-based-intervention

[ref2] Ames M. E. Leadbeater B. J. (2018). Depressive symptom trajectories and physical health: persistence of problems from adolescence to young adulthood. J. Affect. Disord. 240, 121–129. doi: 10.1016/j.jad.2018.07.001 30064077

[ref3] Blewitt C. Fuller-Tyszkiewicz M. Nolan A. Bergmeier H. Vicary D. Huang T. . (2018). Social and emotional learning associated with universal curriculum-based interventions in early childhood education and care centers: a systematic review and meta-analysis. JAMA Netw. Open 1:e185727. doi: 10.1001/jamanetworkopen.2018.5727, PMID: 30646283 PMC6324369

[ref01] Braun V. Clarke V. (2006). Using thematic analysis in psychology. Qualitative Res. Psychol. 3, 77–101. doi: 10.1191/1478088706qp063oa

[ref4] Bröder J. Carvalho G. S. (2019). “Health literacy of children and adolescents: conceptual approaches and developmental considerations” in International Handbook of Health Literacy: Research, Practice and Policy Across the Lifespan. eds. Okan O. Bauer U. Levin-Zamir D. Pinheiro P. Sorensen K. (Bristol: Policy Press), 39–52.

[ref5] Burrow A. L. Ratner K. Porcelli S. Sumner R. (2022). Does purpose grow here? Exploring 4-H as a context for cultivating youth purpose. J. Adolesc. Res. 37, 471–500. doi: 10.1177/0743558420942477

[ref6] Campos L. Dias P. Duarte A. Veiga E. Dias C. C. Palha F. (2018). Is it possible to “find space for mental health” in young people? Effectiveness of a school-based mental health literacy promotion program. Int. J. Environ. Res. Public Health 15:1426. doi: 10.3390/ijerph15071426, PMID: 29986444 PMC6069495

[ref7] Campos L. Dias P. Palha F. Duarte A. Veiga E. (2016). Development and psychometric properties of a new questionnaire for assessing mental health literacy in young people. Univ. Psychol. 15, 61–72. doi: 10.11144/Javeriana.upsy15-2.dppq

[ref8] CASEL . (2020). CASEL’s SEL framework: what are the core competence areas and where are they promoted? Available at:https://casel.org/casel-sel-framework-11-2020/

[ref9] CASEL . (2023). About CASEL. Available at: https://casel.org/about-us/

[ref10] Cefai C. Matsopoulos A. Bartolo P. Galea K. Gavogiannaki M. Zanetti M. A. . (2014). A resilience curriculum for early years and primary schools in Europe: enhancing quality education. Croat. J. Educ. 16, 11–32.

[ref11] Coutinho J. Ribeiro E. Ferreirinha R. Dias P. (2010). Versão portuguesa da escala de dificuldades de regulação emocional e sua relação com sintomas psicopatológicos [The Portuguese version of the difficulties in emotion regulation scale and its relationship with psychopathological symptoms]. Revista de Psiquiatria Clínica 37, 145–151. doi: 10.1590/S0101-60832010000400001

[ref12] Deng J. Zhou F. Hou W. Heybati K. Lohit S. Abbas U. . (2023). Prevalence of mental health symptoms in children and adolescents during the COVID-19 pandemic: a meta-analysis. Ann. N. Y. Acad. Sci. 1520, 53–73. doi: 10.1111/nyas.14947, PMID: 36537131 PMC9880764

[ref13] Durlak J. A. Weissberg R. P. Dymnicki A. B. Taylor R. D. Schellinger K. B. (2011). The impact of enhancing students’ social and emotional learning: a meta-analysis of school-based universal interventions. Child Dev. 82, 405–432. doi: 10.1111/j.1467-8624.2010.01564.x, PMID: 21291449

[ref14] Fleary S. A. Joseph P. Pappagianopoulos J. E. (2018). Adolescent health literacy and health behaviors: a systematic review. J. Adolesc. 62, 116–127. doi: 10.1016/j.adolescence.2017.11.010 29179126

[ref15] Francisco R. (2020). Versão Portuguesa do health literacy for school-aged children [Portuguese version of the health literacy for school-aged children—research version]. Lisboa: Universidade Católica Portuguesa.

[ref16] Francisco R. Hormigo M. Sesifredo M. Raposo B. (2019a). Versão Portuguesa do EPOCH—Medida de bem-estar do adolescente [Portuguese version of the EPOCH measure of adolescent well-being—research version]. Lisboa: Universidade Católica Portuguesa.

[ref17] Francisco R. Raposo B. Hormigo M. Sesifredo M. (2019b). “#EntreViagenseAprendizagens: development of a program for the promotion of wellbeing and healthy lifestyles” in Book of abstracts of the 5th international congress of clinical and Health Psychology with children and adolescents, Oviedo, 14–16 november 2019. eds. Orgilés M. Fernández-Martínez I. (Madrid: Ediciones Pirámide), 91–92.

[ref18] Frech A. (2012). Healthy behavior trajectories between adolescence and young adulthood. Adv. Life Course Res. 17, 59–68. doi: 10.1016/j.alcr.2012.01.003, PMID: 22745923 PMC3381431

[ref19] Frey M. Obermeier V. von Kries R. Schulte-Körne G. (2020). Age and sex specific incidence for depression from early childhood to adolescence: a 13-year longitudinal analysis of German health insurance data. J. Psychiatr. Res. 129, 17–23. doi: 10.1016/j.jpsychires.2020.06.001, PMID: 32554228

[ref20] Froh J. J. Bono G. Fan J. Emmons R. A. Henderson K. Harris C. . (2014). Nice thinking! An educational intervention that teaches children to think gratefully. Sch. Psychol. Rev. 43, 132–152. doi: 10.1080/02796015.2014.12087440

[ref21] Golden T. (2020). Reframing Photovoice: building on the method to develop more equitable and responsive research practices. Qual. Health Res. 30, 960–972. doi: 10.1177/1049732320905564, PMID: 32081060

[ref22] Gratz K. L. Roemer L. (2004). Multidimensional assessment of emotion regulation and dysregulation: development, factor structure, and initial validation of the difficulties in emotion regulation scale. J. Psychopathol. Behav. Assess. 26, 41–54. doi: 10.1023/B:JOBA.0000007455.08539.94

[ref23] Greenberg M. T. Domitrovich C. E. Weissberg R. P. Durlak J. A. (2017). Social and emotional learning as a public health approach to education. Future Child. 27, 13–32. doi: 10.1353/foc.2017.0001

[ref24] Gunnell K. E. Flament M. F. Buchholz A. Henderson K. A. Obeid N. Schubert N. . (2016). Examining the bidirectional relationship between physical activity, screen time, and symptoms of anxiety and depression over time during adolescence. Prev. Med. 88, 147–152. doi: 10.1016/j.ypmed.2016.04.002, PMID: 27090920

[ref25] Hart L. M. Mason R. J. Kelly C. M. Cvetkovski S. Jorm A. F. (2016). “Teen mental health first aid”: a description of the program and an initial evaluation. Int. J. Ment. Heal. Syst. 10, 3–18. doi: 10.1186/s13033-016-0034-1, PMID: 26788123 PMC4717562

[ref26] Hendriks A. M. Bartels M. Stevens G. W. J. M. Walsh S. D. Torsheim T. Elgar F. J. . (2020). National child and adolescent health policies as indicators of adolescent mental health: a multilevel analysis of 30 European countries. J. Early Adolesc. 40, 537–565. doi: 10.1177/0272431619858413

[ref27] Hormigo M. Francisco R. (2019). “Evaluation of #EntreViagenseAprendizagens implemented in two schools: qualitative and quantitative results,” in Book of Abstracts of the 5th International Congress of Clinical and Health Psychology With Children and Adolescents, Oviedo, 14 -16 November 2019. eds. M. Orgilés and I. Fernández-Martínez (Madrid: Ediciones Pirámide), 92.

[ref28] Hsu W. Chiang C. Yang S. (2014). The effect of individual factors on health behaviors among college students: the mediating effects of eHealth literacy. J. Med. Internet Res. 16:e287. doi: 10.2196/jmir.3542, PMID: 25499086 PMC4275503

[ref29] Inchley J. Currie D. Cosma A. Samdal O. (2018). Health behaviour in school-aged children (HBSC) study protocol: Background, methodology and mandatory items for the 2017/18 survey. CAHRU.

[ref30] Irvine D. S. McGarity-Shipley E. Lee E.-Y. Janssen I. Leatherdale S. T. (2022). Longitudinal associations between e-cigarette use, cigarette smoking, physical activity, and recreational screen time in Canadian adolescents. Nicotine Tob. Res. 24, 978–985. doi: 10.1093/ntr/ntab248, PMID: 34850182

[ref31] Jarden A. Jarden R. Chin T. Kern M. L. (2021). “Assessing wellbeing in school communities” in The Palgrave Handbook of Positive Education. eds. Kern M. L. Wehmeyer M. L. (London: Palgrave Macmillan), 297–324.

[ref32] Jorm A. F. (2000). Mental health literacy: public knowledge and beliefs about mental disorders. Br. J. Psychiatry 177, 396–401. doi: 10.1192/bjp.177.5.396 11059991

[ref33] Kashdan T. B. McKnight P. E. Goodman F. R. (2022). Evolving positive psychology: a blueprint for advancing the study of purpose in life, psychological strengths, and resilience. J. Posit. Psychol. 17, 210–218. doi: 10.1080/17439760.2021.2016906

[ref34] Kern M. L. Benson L. Steinberg E. A. Steinberg L. D. (2016). The EPOCH measure of adolescent well-being. Psychol. Assess. 28, 586–597. doi: 10.1037/pas0000201 26302102

[ref35] Kern M. L. Waters L. E. Adler A. White M. A. (2015). A multidimensional approach to measuring well-being in students: application of the PERMA framework. J. Posit. Psychol. 10, 262–271. doi: 10.1080/17439760.2014.936962, PMID: 25745508 PMC4337659

[ref36] Khanna P. Singh K. (2016). Effect of gratitude educational intervention on well-being indicators among north Indian adolescents. Contemp. Sch. Psychol. 20, 305–314. doi: 10.1007/s40688-016-0087-9

[ref37] Kutcher S. Wei Y. Costa S. Gusmão R. Skokauskas N. Sourander A. (2016). Enhancing mental health literacy in young people. Eur. Child Adolesc. Psychiatry 25, 567–569. doi: 10.1007/s00787-016-0867-9 27236662

[ref38] Layous K. Nelson S. K. Oberle E. Schonert-Reichl K. A. Lyubomirsky S. (2012). Kindness counts: prompting prosocial behavior in preadolescents boosts peer acceptance and well-being. PLoS One 7, 7–9. doi: 10.1371/journal.pone.0051380, PMID: 23300546 PMC3530573

[ref39] Leadbeater B. Thompson K. Gruppuso V. (2012). Co-occurring trajectories of symptoms of anxiety, depression, and oppositional defiance from adolescence to young adulthood. J. Clin. Child Adolesc. Psychol. 41, 719–730. doi: 10.1080/15374416.2012.694608, PMID: 22742519 PMC4905756

[ref40] Marques A. Demetriou Y. Tesler R. Gouveia É. R. Peralta M. Matos M. G. (2019). Healthy lifestyle in children and adolescents and its association with subjective health complaints: findings from 37 countries and regions from the HBSC study. Int. J. Environ. Res. Public Health 16:3292. doi: 10.3390/ijerph16183292, PMID: 31500252 PMC6765801

[ref41] Norrish J. M. Vella-Brodrick D. A. (2009). Positive psychology and adolescents: where are we now? Where to from here? Aust. Psychol. 44, 270–278. doi: 10.1080/00050060902914103

[ref42] Oades L. G. Baker L. M. Francis J. J. Taylor J. A. (2021). “Wellbeing literacy and positive education” in The Palgrave Handbook of Positive Education. eds. Kern M. L. Wehmeyer M. L. (London: Palgrave Macmillan), 325–343.

[ref43] Orben A. Lucas R. E. Fuhrmann D. Kievit R. A. (2022). Trajectories of adolescent life satisfaction. R. Soc. Open Sci. 9:211808. doi: 10.1098/rsos.211808, PMID: 35937913 PMC9346371

[ref44] Organisation for Economic Co-operation and Development . (2021). OECD survey on social and emotional skills: technical report. Available at: https://www.oecd.org/education/ceri/social-emotional-skills-study/sses-technical-report.pdf

[ref45] Owens R. L. Waters L. (2020). What does positive psychology tell us about early intervention and prevention with children and adolescents? A review of positive psychological interventions with young people. J. Posit. Psychol. 15, 588–597. doi: 10.1080/17439760.2020.1789706

[ref46] Paakkari L. Paakkari O. (2012). Health literacy as a learning outcome in schools. Health Educ. 112, 133–152. doi: 10.1108/09654281211203411

[ref47] Paakkari O. Torppa M. Kannas L. Paakkari L. (2016). Subjective health literacy: development of a brief instrument for school-aged children. Scand. J. Public Health 44, 751–757. doi: 10.1177/1403494816669639 27655781

[ref48] Paakkari L. Torppa M. Mazur J. Boberova Z. Sudeck G. Kalman M. . (2020). A comparative study on adolescents’ health literacy in Europe: findings from the HBSC study. Int. J. Environ. Res. Public Health 17:3543. doi: 10.3390/ijerph17103543, PMID: 32438595 PMC7277198

[ref49] Paakkari O. Torppa M. Villberg J. Kannas L. Paakkari L. (2018). Subjective health literacy among school-aged children. Health Educ. 118, 182–195. doi: 10.1108/HE-02-2017-0014

[ref50] Peterson C. Seligman M. (2004). Character Strengths and Virtues: A Handbook and Classification. Washington, DC; Oxford: APA Press and Oxford University Press.

[ref51] Pleasant A. Griffin K. H. Maish C. O’Leary C. Carmona R. (2019). “Health literacy interventions for children or adolescents: an overview and insights into practical applications” in International Handbook of Health Literacy. eds. Okan O. Bauer U. Levin-Zamir D. Pinheiro P. Sørensen K. (Bristol: Policy Press), 307–322.

[ref52] Proctor C. Tsukayama E. Wood A. M. Maltby J. Eades J. F. Linley P. A. (2011). Strengths gym: the impact of a character strengths-based intervention on the life satisfaction and well-being of adolescents. J. Posit. Psychol. 6, 377–388. doi: 10.1080/17439760.2011.594079

[ref53] Raich R. M. Sánchez-Carracedo D. López-Guimerà G. Portell M. Moncada A. Fauquet J. (2008). A controlled assessment of school-based preventive programs for reducing eating disorder risk factors in adolescent Spanish girls. Eat. Disord. 16, 255–272. doi: 10.1080/10640260802016852, PMID: 18443983

[ref54] Ross D. A. Hinton R. Melles-Brewer M. Engel D. Zeck W. Fagan L. . (2020). Adolescent well-being: a definition and conceptual framework. J. Adolesc. Health 67, 472–476. doi: 10.1016/j.jadohealth.2020.06.042, PMID: 32800426 PMC7423586

[ref55] Seligman M. Csikszentmihalyi M. (2000). Positive psychology: an introduction. Am. Psychol. 55, 5–14. doi: 10.1037/0003-066X.55.1.5 11392865

[ref56] Seligman M. Ernst R. M. Gillham J. Reivich K. Linkins M. (2009). Positive education: positive psychology and classroom interventions. Oxf. Rev. Educ. 35, 293–311. doi: 10.1080/03054980902934563

[ref57] Silva C. S. Marques A. Mendes R. Silva M. N. Tomás R. Teixeira P. J. (2018). The Portuguese physical activity barometer: perceptions, attitudes, motivation and knowledge. J. Phys. Act. Health 15:S142.

[ref58] Simões C. Santos A. C. Lebre P. Daniel J. R. Branquinho C. Gaspar T. . (2021). Assessing the impact of the European resilience curriculum in preschool, early and late primary school children. Sch. Psychol. Int. 42, 539–566. doi: 10.1177/01430343211025075

[ref59] Skre I. Friborg O. Breivik C. Johnsen L. I. Arnesen Y. Wang C. E. A. (2013). A school intervention for mental health literacy in adolescents: effects of a non-randomized cluster controlled trial. BMC Public Health 13:873. doi: 10.1186/1471-2458-13-873, PMID: 24053381 PMC3850725

[ref60] Steger M. F. O’Donnell M. B. Morse J. L. (2021). “Helping students find their way to meaning: meaning and purpose in education” in The Palgrave Handbook of Positive Education. eds. Kern M. L. Wehmeyer M. L. (London: Palgrave Macmillan), 551–578.

[ref61] Svendsen M. T. Bak C. K. Sørensen K. Pelikan J. Riddersholm S. J. Skals R. K. . (2020). Associations of health literacy with socioeconomic position, health risk behavior, and health status: a large national population-based survey among Danish adults. BMC Public Health 20, 1–12. doi: 10.1186/s12889-020-08498-8, PMID: 32345275 PMC7187482

[ref62] Taylor R. D. Oberle E. Durlak J. A. Weissberg R. P. (2017). Promoting positive youth development through school-based social and emotional learning interventions: a meta-analysis of follow-up effects. Child Dev. 88, 1156–1171. doi: 10.1111/cdev.12864, PMID: 28685826

[ref63] Velten J. Bieda A. Scholten S. Wannemüller A. Margraf J. (2018). Lifestyle choices and mental health: a longitudinal survey with German and Chinese students. BMC Public Health 18, 1–15. doi: 10.1186/s12889-018-5526-2, PMID: 29769115 PMC5956886

[ref64] Wang C. Burris M. A. (1997). Photovoice: concept, methodology, and use for participatory needs assessment. Health Educ. Behav. 24, 369–387. doi: 10.1177/109019819702400309, PMID: 9158980

[ref65] World Health Organization (2013). “Health literacy: the solid facts” in Health literacy: The solid facts. eds. Kickbusch I. Pelikan J. M. Apfel F. Tsouros A. D. (Copenhagen: World Health Organization)

[ref66] World Health Organization . (2021). Health literacy in the context of health, well-being and learning outcomes: the case of children and adolescents in schools. Available at: http://apps.who.int/bookorders.

[ref67] World Health Organization and United Nations Educational, Scientific and Cultural Organization . (2021). Making every school a health-promoting school: global standards and indicators for health-promoting schools and systems. Available at: https://www.who.int/publications/i/item/9789240025059

